# A polysaccharide utilization locus from *Chitinophaga pinensis* simultaneously targets chitin and β-glucans found in fungal cell walls

**DOI:** 10.1128/msphere.00244-23

**Published:** 2023-07-26

**Authors:** Zijia Lu, Alma Kvammen, He Li, Mengshu Hao, Annie R. Inman, Vincent Bulone, Lauren S. McKee

**Affiliations:** 1 Department of Chemistry, Division of Glycoscience, KTH Royal Institute of Technology, Stockholm, Sweden; 2 College of Medicine and Public Health, Flinders University, Adelaide, Australia; 3 Wallenberg Wood Science Center, KTH Royal Institute of Technology, Stockholm, Sweden; University of Georgia, Athens, Georgia, USA

**Keywords:** Bacteroidota, β-1,3-glucan, carbohydrate-binding module, chitin, *Chitinophaga*, glycoside hydrolase, polysaccharide utilization locus

## Abstract

In nature, complex carbohydrates are rarely found as pure isolated polysaccharides. Instead, bacteria in competitive environments are presented with glycans embedded in heterogeneous matrices such as plant or microbial cell walls. Members of the Bacteroidota phylum thrive in such ecosystems because they are efficient at extracting nutrients from complex substrates, secreting consortia of synergistic enzymes to release metabolizable sugars. Carbohydrate-binding modules (CBMs) are used to target enzymes to substrates, enhancing reaction rate and product release. Additionally, genome organizational tools like polysaccharide utilization loci (PULs) ensure that the appropriate set of enzymes is produced when needed. In this study, we show that the soil bacterium *Chitinophaga pinensis* uses a PUL and several CBMs to coordinate the activities of enzymes targeting two distinct polysaccharides found in fungal cell walls. We describe the enzymatic activities and carbohydrate-binding behaviors of components of the fungal cell wall utilization locus (FCWUL), which uses multiple chitinases and one β-1,3-glucanase to hydrolyze two different substrates. Unusually, one of the chitinases is appended to a β-glucan-binding CBM, implying targeting to a bulk cell wall substrate rather than to the specific polysaccharide being hydrolyzed. Based on our characterization of the PUL’s outer membrane sensor protein, we suggest that the FCWUL is activated by β-1,3-glucans, even though most of its enzymes are chitin-degrading. Our data showcase the complexity of polysaccharide deconstruction in nature and highlight an elegant solution for how multiple different glycans can be accessed using one enzymatic cascade.

**IMPORTANCE:**

We report that the genome of the soil bacterium *Chitinophaga pinensis* encodes three multi-modular carbohydrate-active enzymes that work together to hydrolyze the major polysaccharide components found in fungal cell walls (FCWs). The enzymes are all encoded by one polysaccharide utilization locus and are co-expressed, potentially induced in the presence of β-1,3-glucans. We present biochemical characterization of each enzyme, including the appended carbohydrate-binding modules that likely tether the enzymes to a FCW substrate. Finally, we propose a model for how this so-called fungal cell wall utilization locus might enable *C. pinensis* to metabolize both chitin and β-1,3-glucans found in complex biomass in the soil.

## INTRODUCTION

The phylum Bacteroidota (formerly Bacteroidetes) is known to be proficient at the breakdown and metabolism of complex carbohydrates. In diverse glycan-rich environments, the Bacteroidota employ genomic structures called polysaccharide utilization loci (PULs) to control and coordinate the expression of the genes involved in metabolizing a particular polysaccharide (1). A PUL is a discrete cassette of genes that typically encodes the following functional proteins: (i) an outer-membrane glycan-sensing protein that recognizes a particular polysaccharide fragment (SusD-like proteins); (ii) an outer-membrane pore that brings that same fragment into the periplasm (SusC-like proteins); (iii) a glycan sensor-transcriptional regulator system traversing the inner membrane that responds to the polysaccharide fragment to increase expression of genes in the PUL; and (iv) one or more enzymes that, together, can deconstruct the target polysaccharide into low molecular weight metabolizable sugars (1, 2). PULs play a crucial role in coordinating the expression of genes involved in polysaccharide catabolism. The enzymes involved are in most cases glycoside hydrolases (GHs), but accessory enzymes such as carbohydrate esterases are sometimes required for polysaccharide degradation and are often also encoded by PULs (3). The PUL enzymes have complementary activities and can work synergistically to achieve total or near-total saccharification of the target polysaccharides. Biochemical characterization of the enzymes encoded by a PUL can therefore give insight into a species’ metabolism, as well as suggest new biotechnological pathways for biomass deconstruction in industry (4).

**Fig 1:**
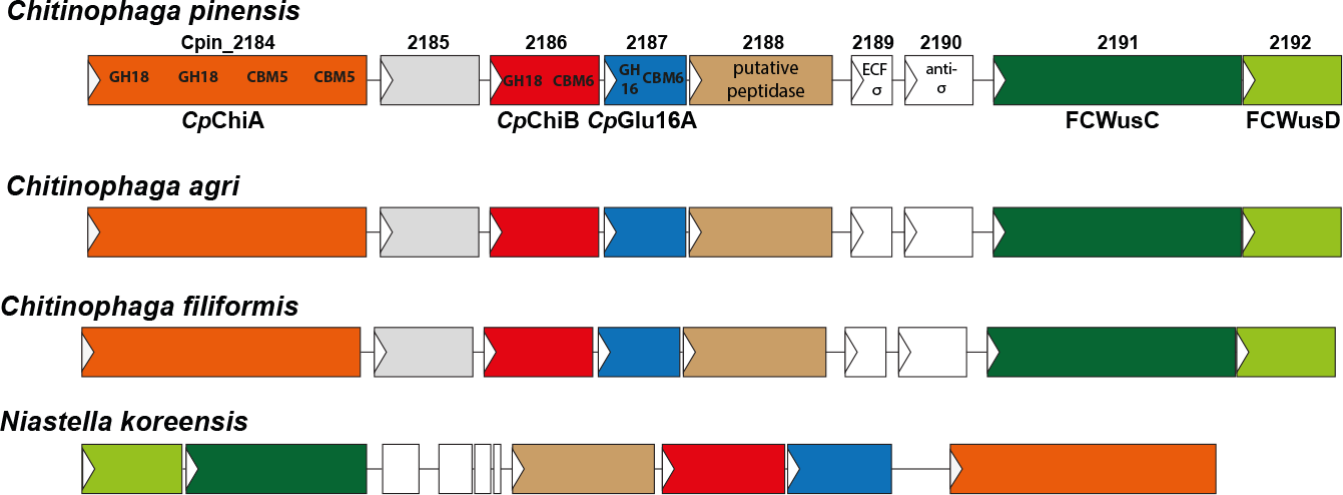
Putative PULs from selected Bacteroidota species. Top, the *Chitinophaga pinensis* FCWUL is proposed to span genes with locus tags *Cpin_2184* to *Cpin_2192*, as shown. The colored genes in the *C. pinensis* locus have equivalents (i.e., the same CAZyme modularity or predicted function) in the other loci depicted, which are shown in the same color. The gene product of *Cpin_2185* is a putative β/γ crystallin lysyl endopeptidase according to KEGG and NCBI automated annotations. *Cpin_2189* and *Cpin_2190* encode an ECF-σ and anti-σ factor, respectively. The arrows on each gene point in the direction of transcription. PULs from other species with full or partial predicted functional synteny to the FCWUL are shown, with color coding matched to the CAZyme architecture of the labeled components of the *C. pinensis* locus. Syntenic PULs were identified using the KEGG server gene cluster analysis tool together with manual NCBI searching.

The environmental Bacteroidota *Chitinophaga pinensis* (5 - 7) encodes a vast number of GHs and other carbohydrate-active enzymes (so-called CAZymes) (8 - 10). Many of these are predicted by family classification at the CAZy database (http://www.cazy.org/) to be chitinases or β-glucanases, and likely target polysaccharides found in fungal cell walls (FCWs), due to the propensity of the bacterium for growing on carbon sources of microbial origin (11 - 13). The genome of *C. pinensis* comprises multiple PULs, although by no means all encoded CAZymes are found within PULs, suggesting at least some reliance on other mechanisms for the regulation of CAZyme gene expression (1, 12). Here, we report the identification and characterization of one putative PUL that bears all the hallmarks of such a locus (Fig. 1), except that the CAZymes encoded are not all predicted to hydrolyze or bind to the same polysaccharide.

The PUL from *C. pinensis* being investigated here contains three genes that encode multi-modular CAZymes and may also include at least one protease/peptidase (Fig. 1). Multi-modularity is common among CAZymes produced by Bacteroidota. Catalytic modules of GH enzymes are often appended to carbohydrate-binding modules (CBMs), whose functional contribution is usually to improve enzyme efficacy by increasing the chances of a productive enzyme-substrate interaction (14). A CBM typically binds to the same polysaccharide that its partner GH is hydrolyzing (15), although there are exceptions to this, such as the xylanase enzymes that are natively appended to cellulose-binding CBMs (16). In such cases, the CBM may help to target the GH to bulk cell wall material, so the targeting/proximity effect still applies (17, 18).

All of the CAZyme genes in the PUL discussed here encode GHs appended to CBMs. In addition, all are predicted to function extracellularly because they all carry standard bacterial SpI signal peptides (19 - 21) and all but one carry the C-terminal domain for secretion via the type IX secretion system, T9SS (22). Specifically, the protein encoded by *Cpin_2184* contains two putative chitinase domains from GH family 18 and two putative chitin-binding domains from CBM family 5. The *Cpin_2186* gene product contains one GH18 putative chitinase and a binding domain from family CBM6, for which binding to diverse polysaccharides has been observed (23 - 28), but chitin binding has not. Finally, the *Cpin_2187* gene product contains a GH16 putative β-glucanase and another CBM6 domain. The combination of modules within these proteins, and the diversity of predicted activities within the PUL, suggests that the locus is targeting more than one polysaccharide. Thus, we pursued full biochemical characterization of the GHs and CBMs, and investigated the glycan binding specificity of the SusD-like protein (*Cpin_2192*) to gain insight into the PUL’s likely activating glycan (1).

We demonstrate that the GH18 enzymes contribute to the deconstruction of chitin, and that the GH16 enzyme can break down β-1,3-glucans (13). As these polysaccharides are key components of FCWs, a known carbon source for *C. pinensis* (12), we propose that this PUL is dedicated to the deconstruction of complex cell wall material, and should be referred to as a fungal cell wall utilization locus (FCWUL). Enzyme synergy in the FCWUL not only applies to the use of three distinct chitinase domains to target the recalcitrant chitin polymer but also involves the concomitant deconstruction of other cell wall polymers, namely β-1,3-glucans and perhaps even the structural proteins that comprise up to 30% dry weight of the FCW in certain fungi (29). The characterization of the FCWUL has implications for the use of *C. pinensis* in industrial biotechnology for the deconstruction of fungal biomass and for understanding the ecosystem impacts of this bacterium in a fungus-rich natural environment (30).

## RESULTS AND DISCUSSION

The GH and CBM domains encoded by genes *Cpin_2184*, *Cpin_2186*, *Cpin_2187*, and *Cpin_2192* were produced recombinantly, as shown in Fig. S1. Based on the CAZy family annotation of these proteins, it was expected that the GH18 modules of Cpin_2184 and Cpin_2186 would show chitinase activity, while the GH16 module of Cpin_2187 would likely function as a β-glucanase. The binding specificities of the CBMs and the SusD-like protein (Cpin_2192) were less predictable; although chitin-binding has been observed for CBM5 (31, 32), and β-1,3-glucan binding has been observed for CBM6 (27), a wide range of potential ligands were screened for all putative binding modules.

### Cpin_2184 encodes a large protein with complementary chitin-binding and chitin-hydrolyzing domains

The product of gene *Cpin_2184* is a multi-modular protein of approximately 145 kDa, with a complex domain architecture. The enzyme contains two putative chitinase domains from family GH18, two putative chitin-binding CBM5 domains, and three Bacterial Ig-like domains (Pfam 17957). The C-terminal GH18 was previously shown to be a non-processive *exo*-chitinase (33). Although the domain architecture differs, this protein is somewhat analogous to the multi-modular *Fj*ChiA enzyme from the related environmental species *Flavobacterium johnsoniae* that Larsbrink et al. characterized, showing that it utilizes two synergistic GH18 modules with distinct modes of action (*endo* vs *exo*), and at least one non-catalytic chitin-binding domain, to achieve powerful deconstruction of crystalline chitin (34, 35). The full-length multi-modular product of gene *Cpin_2184* will hereafter be referred to as *Cp*ChiA.

The N-terminal GH18 domain (*Cp*ChiA_N_) was produced in a recombinant form that contained only the catalytic module. The enzyme was screened for activity on three chitin substrates, and released a small amount of reducing sugars from each of them (Fig. S2A). However, the activity was very low, and there was not sufficient release of reaction products for the kinetics or mode of action of *Cp*ChiA_N_ to be determined.

**Fig 2:**
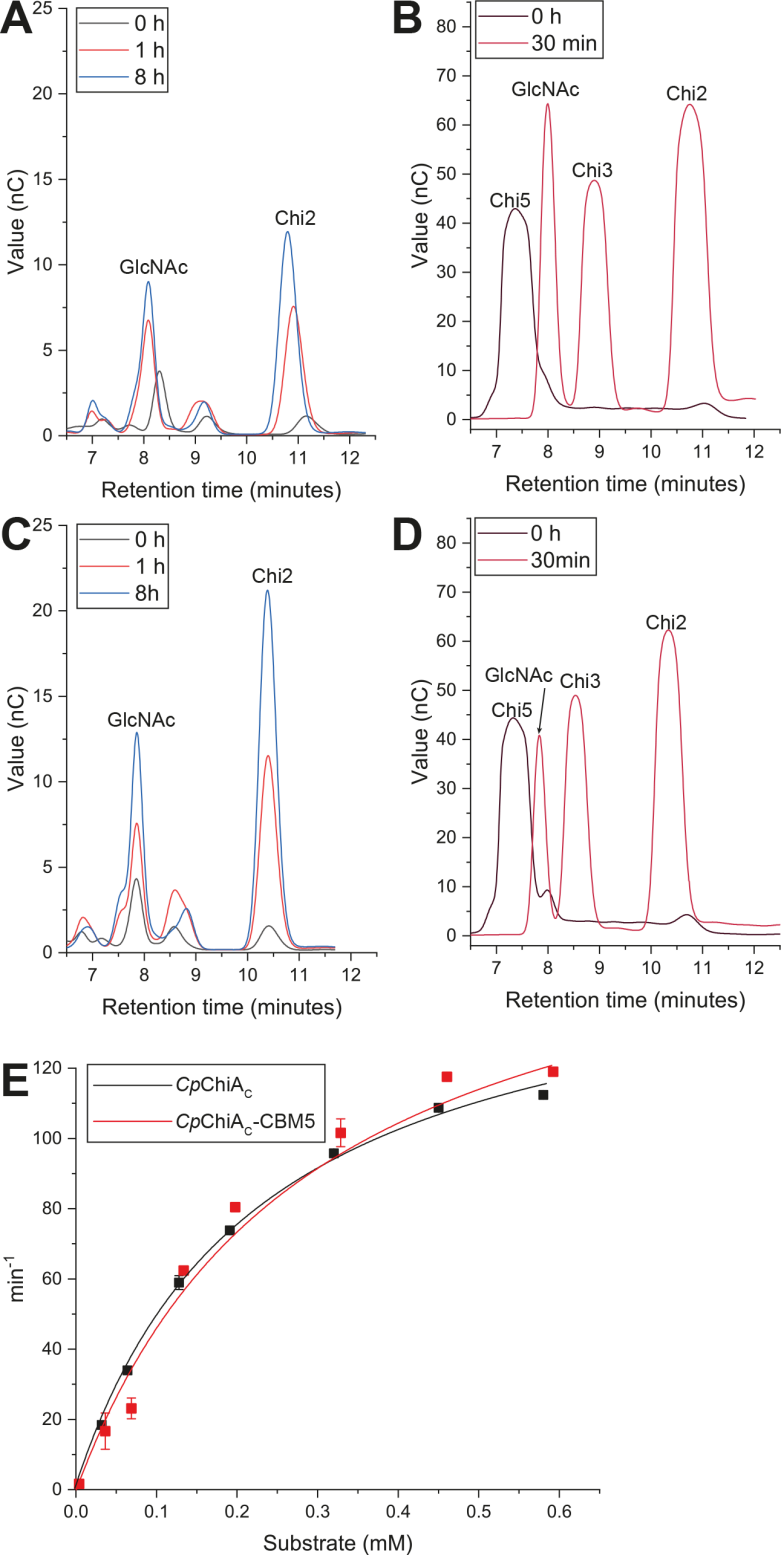
Chitinase activity of *Cp*ChiA. *Exo*-chitinase activity of *Cp*ChiA_C_ (**A, B**) and *Cp*ChiA_C_-CBM5 (**C, D**) is demonstrated via HPAEC-PAD analysis of hydrolytic products generated during incubation with β-chitin (**A, C**). GlcNAc and Chi2 are the major products from this substrate, and the product profile does not change over time, indicating an *exo* mode of hydrolysis. The same final products are released from the pentasaccharide substrate Chi5 (**B, D**). (**E**) Kinetic analysis of *Cp*ChiA_C_ and *Cp*ChiA_C_-CBM5 against the 4MU-Chi3 fluorescent substrate.

Two protein variants containing the C-terminal GH18 domain were produced; *Cp*ChiA_C_ contained only the catalytic module, while *Cp*ChiA_C_-CBM5 contained a series of putative binding domains, namely two Ig-like modules and two CBM5 domains. Echoing previous results (33), this C-terminal catalytic domain was confirmed in our experiments to have an *exo*-mode of action, releasing GlcNAc and chitobiose (Chi2) as the major products from both polymeric and oligomeric chitin substrates (Fig. 2A–D). Kinetic properties of *Cp*ChiA_C_ and *Cp*ChiA_C_-CBM5 were assessed using a fluorescently labeled form of chitotriose (4MU-Chi3) and were highly similar for both forms of the protein, as shown in Fig. 2E and Table 1. However, the modular protein *Cp*ChiA_C_-CBM5 showed higher activity on crystalline chitin (Fig. S2), perhaps due to enhanced substrate interactions via the binding domains. Indeed, binding to polymeric chitin by *Cp*ChiA_C_-CBM5 was verified by pull-down assay, which showed no binding for *Cp*ChiA_C_ alone, confirming that the CBM domain(s) drive this interaction (Fig. S3). No binding to soluble chito-oligosaccharides (ChiOs) could be detected in experiments based on isothermal titration calorimetry (data not shown), suggesting that the CBM5 domains bind preferentially to high molecular weight ligands. This indicates that binding interactions with chitin polymer improve the enzyme’s ability to productively interact with higher molecular weight forms of the substrate. Similar observations were previously made for *Fj*ChiA, which showed an increase in hydrolytic efficiency against crystalline chitin when a domain that binds insoluble polysaccharides was included in the protein (34).

**TABLE 1:** Kinetic parameters of enzymes investigated in this study^*a*^

Enzyme	Substrate	*k*_cat_	*K*_M_	*k*_cat_/*K*_M_	Assay type
*Cp*ChiA_N_	4MU-Chi3	n.d.	n.d.	n.d.	Chitinase assay kit
*Cp*ChiA_C_	4MU-Chi3	159.52 ± 2.1 min^−1^	0.22 ± 0.003 mM	709.03	Chitinase assay kit
*Cp*ChiA_C_-CBM5	4MU-Chi3	181.71 ± 2.3 min^−1^	0.29 ± 0.0002 mM	635.29	Chitinase assay kit
*Cp*ChiB	4MU-Chi3	131.94 ± 8.9 min^−1^	0.24 ± 0.01 mM	530.87	Chitinase assay kit
*Cp*ChiB-CBM6	4MU-Chi3	60.31 ± 3.2 min^−1^	0.12 ± 0.003 mM	489.12	Chitinase assay kit
*Cp*Glu16A-CBM6	Curdlan	2.58 ± 0.2 min^−1^	6.89 ± 1.6 g/L	0.37	DNSA

^*a*^ n.d. denotes that no activity was detected. The chitinase activity of *Cp*ChiA_N_ was too low to be studied kinetically. Error symbols (±) indicate standard error of the mean.

### Cpin_2186 encodes an additional chitinase enzyme with an appended β-glucan-binding CBM

**Fig 3:**
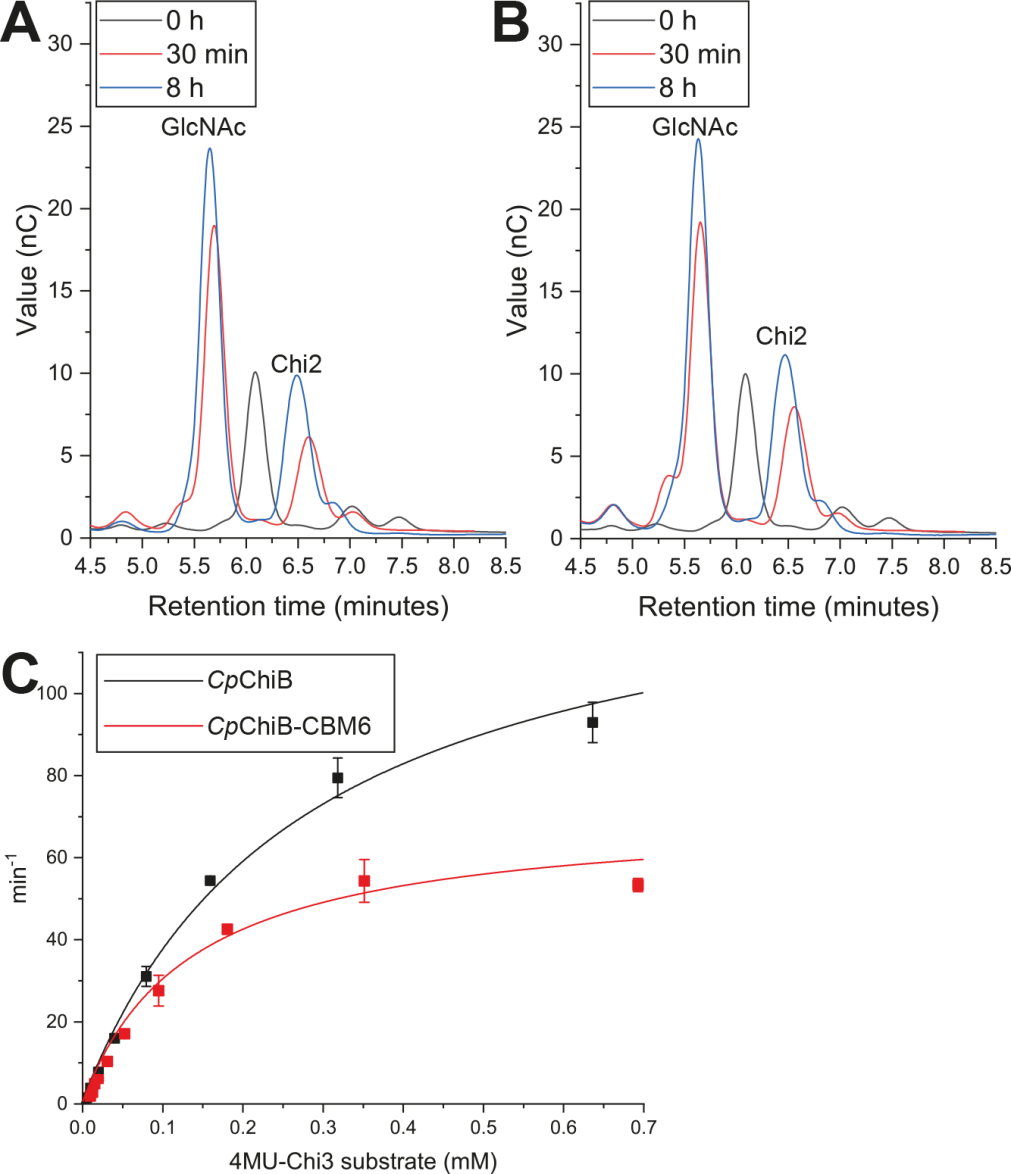
Chitinase and chitin-binding activities of *Cp*ChiB. *Exo*-chitinase activity of *Cp*ChiB (**A**) and *Cp*ChiB-CBM6 (**B**) is demonstrated after HPAEC-PAD analysis of hydrolytic products released from β-chitin after incubation for 30 min or up to 8 h. The product profile does not change over time, indicating an *exo* mode of hydrolysis. (**C**) Kinetic analysis of *Cp*ChiB and *Cp*ChiB-CBM6.

The second chitinase in the PUL is encoded by gene *Cpin_2186*, and has a simpler architecture than *Cp*ChiA, as it comprises just a GH18-predicted chitinase enzyme and a CBM6 putative β-glucan binder. The chitinase domain was produced both with (*Cp*ChiB-CBM6) and without (*Cp*ChiB) its appended binding domain, to compare activity and performance. The enzyme was confirmed to be a chitinase, active on β-chitin and shrimp shell chitin (Fig. S2A), and displayed an apparent *exo* mode of action, releasing GlcNAc and Chi2 as the major products from polymeric chitin (Fig. 3A and B). Kinetic properties were determined using the 4MU-Chi3 substrate (Fig. 3C; Table 1). When a GH enzyme is appended to a CBM, in most cases the CBM binds to the same polysaccharide as the GH hydrolyzes. This often leads to an increase in reaction rate for the enzyme, as proximity to the substrate is enhanced, leading to more productive encounters (14). Indeed, this was observed for *Cp*ChiA_C_ (discussed above): while the rate of reaction on ChiOs was unchanged, the inclusion of the CBM5 domains improved hydrolysis of polymeric chitins, the binding target of the CBMs. However, a binding assay revealed that *Cp*ChiB-CBM6 binds to diverse β-glucans containing β-1,3-linkages, while the catalytic domain alone showed no binding, confirming that this interaction is driven by the CBM (Fig. S3). From this, we can surmise that the CBM6 domain in *Cp*ChiB would not be able to potentiate enzymatic cleavage of chitin when provided as an isolated substrate. In fact we see a catalytic penalty reflected in *k*_cat_ and *k*_cat_/*K*_M_ (Fig. 3; Table 1). We speculate that the binding domain could help localize *Cp*ChiB to a β-glucan-rich chitinous substrate such as an intact FCW, potentiating catalytic activity, but may rather interfere with adherence to chitin substrates that lack β-glucan.

### Cpin_2187 encodes a β-1,3-glucanase with an appended β-glucan-binding CBM

**Fig 4:**
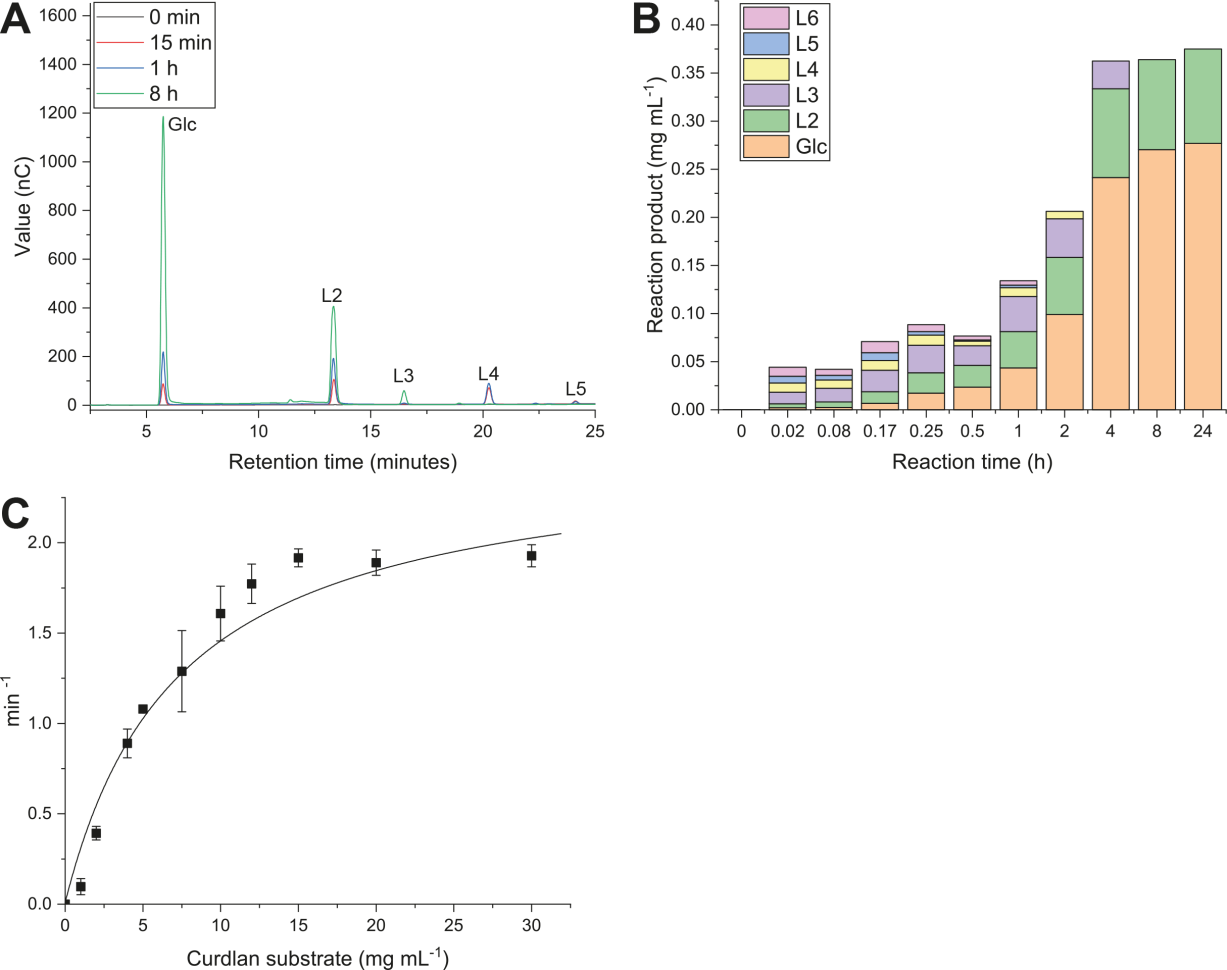
*Endo* β-1,3-glucanase activity of *Cp*Glu16A on curdlan. (A and **B** *) Endo-*glucanase activity of *Cp*Glu16A-CBM6 is demonstrated by HPAEC-PAD analysis of products released from curdlan after incubation from 15 min to 8 h. Longer oligosaccharides (L3–L6) are produced in the first few minutes and are then hydrolyzed, leading to the final products of Glc and L2. (**C**) Kinetic analysis of *Cp*Glu16A-CBM6 activity.

The bi-modular GH16-CBM6 protein encoded by gene *Cpin_2187* was assayed against a range of polysaccharides and found to exclusively act on glucans containing the Glc-β-1,3-Glc linkage (Fig. S2B). The highest activity was observed on curdlan, a linear β-1,3-glucan. Other polysaccharides that partly comprise the same linkage—namely lichenan, laminarin, and mixed linkage barley β-glucan—showed around 80% of the activity seen for curdlan. As the enzyme shows clear β-1,3-glucanase specificity, it is referred to as *Cp*Glu16A-CBM6. Reaction products from the hydrolysis of curdlan were analyzed by high-performance anion-exchange chromatography with pulsed amperometric detection (HPAEC-PAD), to determine the enzyme’s mode of action. After 15 min of incubation, peaks corresponding to glucose were discernible, as well as β-1,3-linked laminari-oligosaccharides of a degree of polymerization (d.p.) 2–6 (Fig. 4A). As the reaction continued for up to 8 h, these longer oligosaccharides were fully hydrolyzed to glucose and the disaccharide L2. No further hydrolysis of L2 was seen up to 24-h incubation (Fig. 4B). These results suggest that *Cp*Glu16A-CBM6 is an *endo*-acting β-1,3-glucanase, able to hydrolyze trisaccharides and longer substrates. Notably, our preliminary screen showed activity on mixed linkage barley β-glucan, where single β-1,3-Glc linkages are found at regular intervals along a chain otherwise composed of β-1,4-Glc linkages. Kinetic parameters for the hydrolysis of curdlan were determined using a reducing sugar assay (Fig. 4C; Table 1). The binding specificity of the CBM domain was investigated in a pull-down assay using a range of insoluble polysaccharides. Because the GH domain consistently hydrolyzed β-1,3-glucans into soluble fragments that could not be pelleted, this assay was performed for a shorter time and with incubation at 4°C, to minimize hydrolysis. It was found that the protein binds to diverse β-glucans including curdlan, lichenan, scleroglucan, pustulan, and yeast β-glucan (Fig. S3). It is likely that this binding specificity of the CBM helps promote enzyme-substrate interactions for the enzyme moiety of *Cp*Glu16A.

### The SusD-like protein encoded by Cpin_2192 binds to β-1,3-glucans

A pair of SusC/D proteins is a conserved feature of the PUL system: SusD-like proteins bind specific glycans in the environment and SusC-like proteins transport them into the periplasm (2, 36). Characterization of the ligand-binding specificity of a recombinant SusD-like protein can therefore give a strong indication of the glycan structure that activates PUL expression (1). In the FCWUL, *Cpin_2191* encodes the SusC-like protein and *Cpin_2192* encodes the SusD-like protein, which we respectively refer to as FCWusC and FCWusD. The FCWusD protein was produced recombinantly, and subjected to a screen for binding against a broad panel of polysaccharides, including but not limited to the substrates of the enzymes in the FCWUL. Figure S3 shows that the protein binds to a range of d-glucans that contain β-1,3 linkages; specifically, there is binding to curdlan, yeast β-glucan, lichenan, and barley β-glucan. The protein does not bind to d-glucans solely comprising either the β-1,4 linkage (cellulose) or the β-1,6 linkage (pustulan), or to any non-glucan polysaccharides tested, confirming linkage specificity. This indicates that upregulation of gene expression in the FCWUL is likely induced in the presence of β-1,3-Glc linkages, a major component in the cell walls of fungi and oomycetes.

### Enzyme synergy in the FCWUL

We propose that the enzymes we have characterized work together to deconstruct the network of complex carbohydrates found in the FCW. Both *Cp*ChiA_C_ and *Cp*ChiB displayed an apparent *exo* mode of action in deconstructing chitin, while the mechanism could not be determined for *Cp*ChiA_N_ due to low *in vitro* activity. If we theorize a synergistic approach for these enzyme domains analogous to that described for the *F. johnsoniae* ChiUL (34), we might presume *Cp*ChiA_N_ to show an *endo* approach to chitin hydrolysis. All the chitinases from the FCWUL were assayed at equimolar concentrations, either alone or in combination, on β-chitin to investigate the potential for synergy deriving from simultaneous activity (Table S1A). After a 24-h incubation with 5 µM enzyme, only *Cp*ChiA_C_-CBM5 released a measurable amount of reducing sugars, reaching a conversion rate of ~2%, and up to 5% conversion after 48 h. In these conditions, there was no detectable activity from *Cp*ChiA_N_ or *Cp*ChiB-CBM6, which typically needed to be incubated for longer or at higher concentrations for activity on this recalcitrant substrate to be measurable. However, when either or both enzymes were incubated together with *Cp*ChiA_C_-CBM5, significantly more reducing sugars were released in 24 h than by *Cp*ChiA_C_-CBM5 alone (Table S1A). Although total chitin saccharification was low, these data indicate that synergistic chitin deconstruction is possible from the chitinases of the FCWUL, even when two enzymes known to be *exo-*acting are provided. It is particularly interesting that even *Cp*ChiA_N_ gives a boost to hydrolysis, despite the very low activity we observed from this enzyme in all other assays.

**Fig 5:**
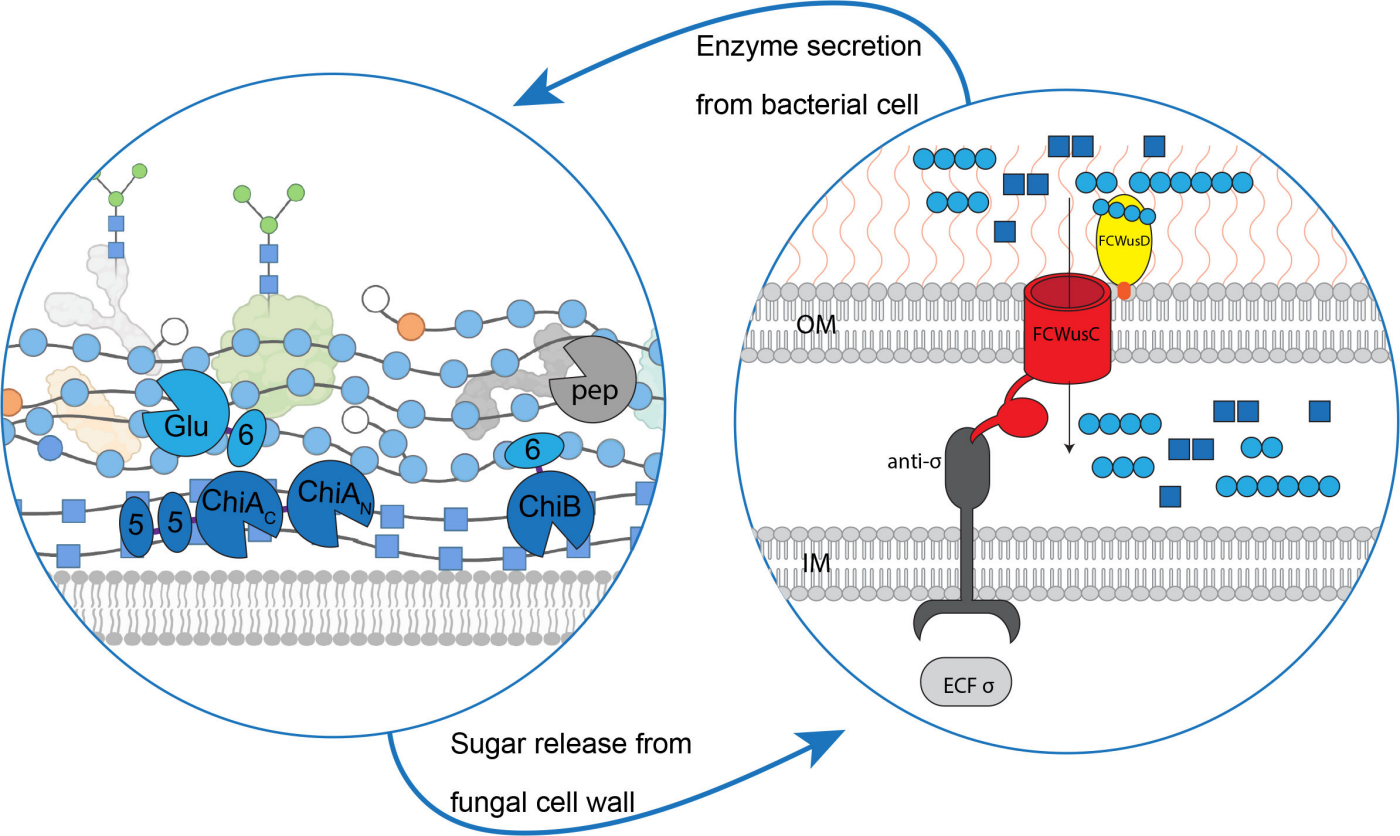
Proposed pathway of synergistic action by the proteins of the FCWUL. Left panel: model of a fungal cell wall undergoing hydrolysis by FCWUL enzymes. Right panel: theoretical model of the FCWUL (OM, outer membrane of bacterial cell; IM, inner membrane of bacterial cell). Components are not shown to scale in either panel. All FCWUL enzymes are depicted as being extracellular, because they all carry standard bacterial SpI signal peptides (19 - 21) and (except for *Cp*Glu16A) the C-terminal domain for secretion via the type IX secretion system, T9SS (22). Dark blue squares, GlcNAc; light blue circles, β-1,3-linked Glc; orange circles, β-1,6-linked Glc; white circles, terminal Glc; green circles, mannose found on glycosylated FCW proteins.

Building on this, we investigated the abilities of the FCWUL chitinases, both in the presence and absence of the β-1,3-glucanase *Cp*Glu16A-CBM6, to break down FCW extracted in-house from button mushrooms (*Agaricus bisporus*) (37). Our data show that *Cp*Glu16A-CBM6 could release a just-detectable amount of reducing sugars, while all the chitinases together could release around 10 times more product (Table S1B). Incubating the FCW substrate with all chitinases plus *Cp*Glu16A-CBM6 led to a slightly more than additive release of reducing sugars from the FCW (Table S1). Despite the low overall substrate conversion efficiency, this points to there being some synergistic activity in these conditions, although our assays used an FCW substrate that was highly compressed and aggregated due to the extraction procedures utilized. A hypothetical model of the mode of action of PUL Cpin_2184-2192 is shown in Fig. 5. When β-1,3-glucan binds to the FCWusD protein, the FCWusC pore is opened and oligosaccharides can enter. This leads to an increase in expression of the CAZymes encoded by the FCWUL. *Cp*ChiA uses two GH18 domains and two chitin-binding CBM5 domains to hydrolyze chitin. It is aided by *Cp*ChiB, which carries a β-1,3-glucan-binding CBM6 domain for tethering to the FCW. *Cp*Glu16A hydrolyzes Glc-β-1,3-Glc linkages, with the help of a β-1,3-glucan binding CBM6 domain. These enzyme activities release Glc- and GlcNAc-based oligosaccharides that can be brought into the periplasm for further metabolism. A putative peptidase (labeled “pep” in Fig. 5) encoded by the FCWUL may be involved in hydrolyzing structural proteins in substrate biomass.

## MATERIALS AND METHODS

### Carbohydrate polymers

A range of carbohydrate polymers was used for testing enzyme activity and protein binding. Chitin crudely extracted from shrimp shells was purchased from Sigma-Aldrich (Darmstadt, Germany). Chitosan, α-chitin, and β-chitin were obtained from Maharani Chitosan PTV, Ltd (Gujarat, India), while scleroglucan (Actigum) was obtained from Cargill (Düsseldorf, Germany). Pustulan was purchased from Biosynth (Berkshire, UK), while microcrystalline cellulose (Avicel), starch, carboxymethylcellulose, and birchwood xylan were purchased from Sigma-Aldrich. Barley β-glucan, konjac glucomannan, curdlan, laminarin, and lichenan were purchased from Bray, Co. Wicklow, Ireland.

### Production and analysis of recombinant proteins

#### Gene cloning

Genes encoding the protein domains under investigation were synthesized in a proprietary vector by ThermoFisher GeneArt, Stockholm, Sweden. Genes were then sub-cloned into the expression vector pET21a (ThermoFisher, Stockholm, Sweden), which carries a C-terminal His_6_ tag and confers ampicillin resistance.

#### Gene expression and protein purification

Plasmids containing a gene of interest were transformed into *Escherichia coli* BL21 (DE3) (Life Technologies, Stockholm, Sweden) by heat shock at 42°C for 30 s. Cells were grown at 37°C with shaking in selective Luria-Bertani (LB) medium containing 50 µg mL^−1^ ampicillin for 2–3 h until an approximate OD_600_ was reached. At this point, gene expression was induced by the addition of 0.2 mM isopropyl-β-d-1-thiogalactopyranoside and the temperature was lowered to 16°C. Protein production proceeded for ~16 h. The cells were then collected by centrifugation at 6,000× *g* for 10 min, resuspended in TALON Buffer A (50 mM sodium phosphate pH 7.4 with 300 mM sodium chloride), lysed by sonication, and the resulting debris were pelleted by centrifugation at 17,000× *g* for 30 min. The supernatant liquid was collected and filtered using a 0.2-µm filter. Recombinant His_6_-tagged proteins were purified using the TALON resin for immobilized metal-ion affinity chromatography, according to the manufacturer’s instructions (Cytiva, Uppsala, Sweden). Unbound or loosely bound non-target proteins were washed from the TALON resin using TALON Buffer B (buffer A with 7.5 mM imidazole) and eluted using TALON Buffer C (buffer A containing increasing concentrations of imidazole, namely 37.5, 75, and 150 mM). Eluted proteins were concentrated, and the buffer was exchanged into 50 mM sodium phosphate pH 6.0 using Amicon Ultra centrifugal filters with a molecular weight cutoff of 3 kDa (Millipore, Darmstadt, Germany). The production and purification of recombinant proteins, as well as their apparent molecular weight, were verified by SDS-PAGE analysis.

### Enzyme activity assays

#### Reducing sugar assays after polysaccharide hydrolysis

Hydrolysis of various polysaccharides was studied using the 3,5-dinitrosalicylic acid (DNSA) reducing sugar assay (38). See “Carbohydrate polymers” section for a full list of substrates and their sources. Substrates at 0–30 g L^−1^ were incubated with the hydrolytic enzymes at 10–100 nM, in 50 mM sodium phosphate buffer, pH 6.5. Hydrolysis of the substrates was measured as an increase in reducing sugars detected by the DNSA assay, using a standard curve of glucose. To this end, samples of enzymatic reactions were added to an equal volume of DNSA reagent (1% [wt/vol] DNSA, 0.2% [vol/vol] phenol, 1% [wt/vol] NaOH, 0.005% [wt/vol] glucose, and 0.05% [wt/vol] Na_2_SO_3_) to terminate the reactions, and the color was developed by boiling the mixtures for 20 min and cooling on ice for 5 min, prior to measuring the absorbance at 575 nm on a Cary 50 spectrophotometer.

#### Detection of chitinase activity using fluorescently labeled oligosaccharides

A chitinase assay kit (CS1030, Sigma-Aldrich) was used for kinetic analysis of the hydrolysis of the fluorescently-labeled chitin oligosaccharide 4-methylumbelliferyl N,N′,N″-triacetyl-β-d-chitotrioside (4MU-Chi3). The kit was used for assays performed in 96-well plates, following the manufacturer’s instructions. Enzymatic hydrolysis releases 4-methylumbelliferone, which is quantified using excitation and emission wavelengths of 360 and 450 nm, respectively.

#### Reaction product analysis by HPAEC-PAD

HPAEC-PAD analysis of oligosaccharides was performed using a Dionex ICS-3000 high-performance liquid chromatography system operated by Chromeleon software version 6.80 (Dionex) using a Dionex CarboPac PA1 column. Solvent A was water, solvent B was 1 M sodium hydroxide, solvent C was 200 mM sodium hydroxide with 170 mM sodium acetate, and solvent D was 1 M sodium acetate. Depending on the analytes, different gradients were employed. For the detection of gluco-oligosaccharides, the following gradient was used: prewash and column calibration, −5 to 0 min 15% B (0.5 mL min^−1^); sample injection, 0–16 min 15% B (0.5 mL min^−1^); gradient elution, 15–30 min 33% B (0.5 mL min^−1^), 30–31 min 33% B and 50% D (0.5 mL min^−1^); and column wash and final elution, 31–35 min 15% B (0.5 mL min^−1^). For the detection of ChiOs, the following gradient was used: prewash and column calibration, −10 to 0 min 15% B (0.5 mL min^−1^); sample injection, 0–15 min 15% B (0.5 mL min^−1^); gradient elution, 15–17 min 15% B and 50% D (0.5 mL min^−1^); and column wash and final elution, 17–18 min 15% B (0.5 mL min^−1^). Carbohydrates were identified and quantified by comparing their retention times and peak areas to those of standards of known concentrations.

### Carbohydrate-binding assays

#### Pull-down assays

Proteins were screened for the capacity to bind insoluble or semi-soluble polysaccharides using a pull-down assay (39). Briefly, 900 µL of polysaccharide at 5 g L^−1^ was incubated with protein at ~0.5–3 g L^−1^ in 50 mM sodium phosphate buffer pH 6.0 for 3 h at room temperature. The mixtures were incubated for 3 h on a Stuart Rotator Disk turning at 24 rpm and centrifuged at 10,000× *g* for 5 min. The supernatants were collected without disturbing the pellets and analyzed by SDS-PAGE. The absence of protein in the supernatants indicates binding to the insoluble polysaccharides, which formed a pellet during centrifugation.

## SUPPLEMENTAL MATERIAL

Supplemental Information msphere.00244-23-s0001.docx Figures and tables providing data supporting the paper.

ASM does not own the copyrights to Supplemental Material that may be linked to, or accessed through, an article. The authors have granted ASM a non-exclusive, world-wide license to publish the Supplemental Material files. Please contact the corresponding author directly for reuse.
