# Supplementary material for: A polysaccharide utilization locus from Chitinophaga pinensis simultaneously targets chitin and β-glucans found in fungal cell walls
Source: mSphere. 2023 Jul 26;8(4):e00244-23. doi: 10.1128/msphere.00244-23 (PMC10449523; doi:10.1128/msphere.00244-23)
Supplement: Supplemental Information — Figures and tables providing data supporting the paper. [file msphere.00244-23-s0001.docx]

# Supplementary Information


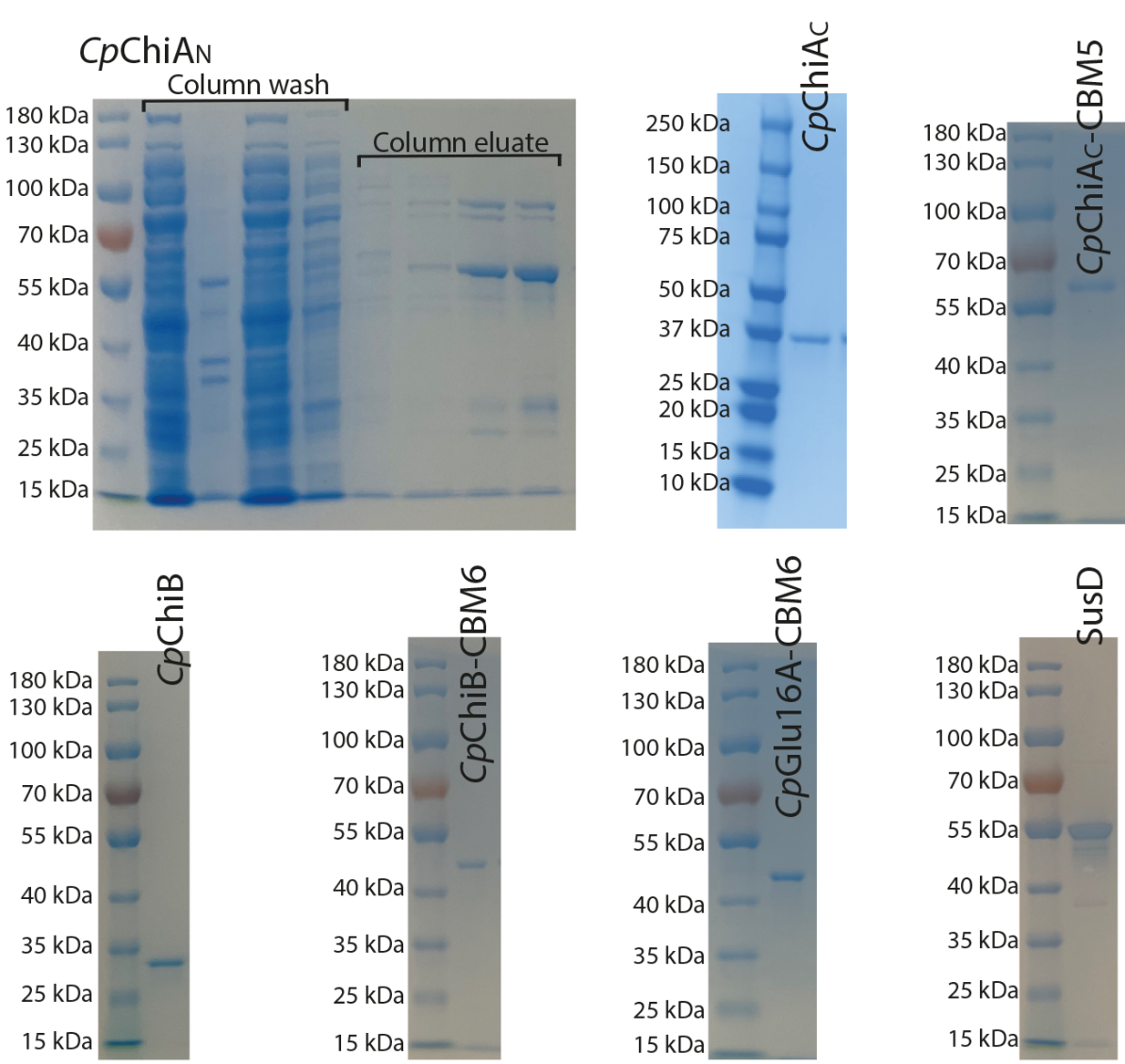


**Supplementary Figure S1. Production and purification of the proteins investigated.** The SDS-PAGE images show a representative example outcome of IMAC purification of the proteins investigated in this study. The first image shows a series of column wash samples and column elutions generated during purification, for reference to the purification method used. Protein of the correct size was obtained in column eluate fractions, which were pooled and washed to remove NaCl and imidazole. For other images, only the final purified protein is shown


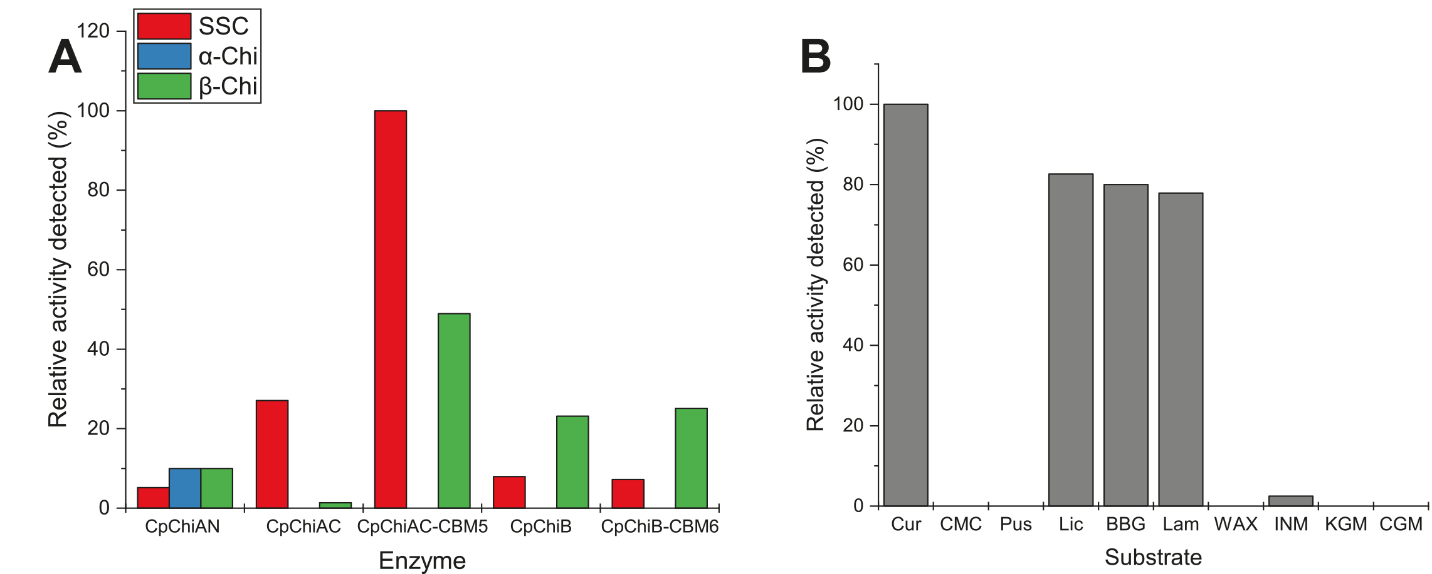


**Supplementary Figure S2. Initial activity screening of the enzymes encoded by the FCWUL. A.** Relative hydrolytic activity against α-chitin (α-Chi), β-chitin (β-Chi), and shrimp shell chitin (SSC) in an overnight incubation with the chitinases encoded by the FCWUL. The relative amounts of reducing sugar released are compared to the reaction of CpChiA_C_-CBM5 against shrimp chitin. **B.** *Cp*Glu16A-CBM6 was screened for activity on a range of polysaccharides and was determined to be a β-glucanase acting preferentially on the linear β-1,3-glucan curdlan and other polysaccharides that contain β-1,3-glucosyl linkages. The polysaccharides used for *Cp*Glu16A-CBM6 screening were as follows: Cur, curdlan; CMC, carboxymethycellulose; Pus, pustulan; Lic, lichenan; BBG, barley β-glucan; Lam, laminarin; WAX, wheat arabinoxylan; INM, ivory nut mannan; KGM, konjac glucomannan; CGM, carob galactomannan.


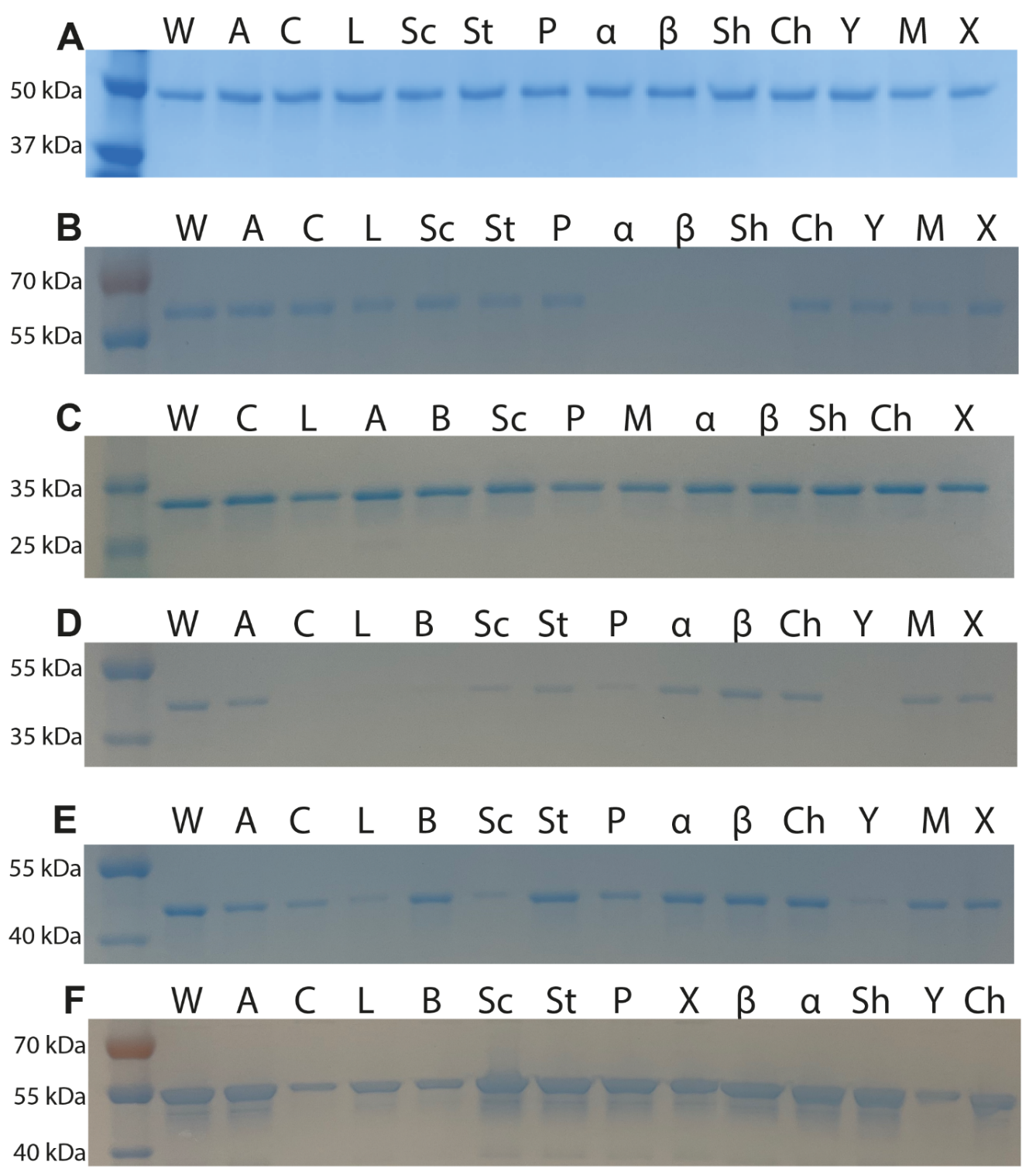


**Supplementary Figure S3. Polysaccharide binding behaviours of the proteins found in the FCWUL.** Pull-down assays were performed using a range of insoluble and semi-soluble polysaccharides to determine the ligand-binding preferences of proteins investigated in this study. If a protein is absent or the intensity of the corresponding band is reduced, this indicates binding to the tested polysaccharides. These gels show representative results of experiments that were repeated at least three times. The polysaccharides used in each sample were as follows: W, water control (no polysaccharide present); A, Avicel cellulose; C, curdlan; L, lichenan; B, barley β-glucan; Sc, scleroglucan; St, starch; P, pustulan; α, α-chitin; β, β-chitin; Sh, shrimp shell chitin; Ch, chitosan; Y, yeast β-glucan; M, ivory nut mannan; X, oat spelt xylan. **A.** *Cp*ChiA_C_ alone does not bind to any polysaccharide. **B.** *Cp*ChiA_C_-CBM5 binds to all forms of chitin but not to chitosan or other polysaccharides. **C.** *Cp*ChiB alone does not bind to any polysaccharide. **D.** *Cp*ChiB-CBM6 binds to lichenan, barley β-glucan, and yeast β-glucan. There is weaker binding to scleroglucan and pustulan. **E.** *Cp*Glu16A-CBM6 binds to curdlan, lichenan, scleroglucan, and yeast β-glucan, with possible weak binding to pustulan. **F.** The SusD-like protein of the FCWUL shows binding to curdlan, lichenan, barley β-glucan, and yeast β-glucan.

**Supplementary Table S1. Synergistic action by the proteins of the FCWUL.** This table shows the percentage of maximum product release, as measured by DNSA reducing sugar assay, and not the total conversion of substrate to hydrolysis product. Data are provided for all experiments after incubation for up to 48 hours. All reactions were performed in triplicate. **A.** Deconstruction of chitin by three GH18 chitinase enzymes, *Cp*ChiA_N_, *Cp*ChiA_C_-CBM5, and *Cp*ChiB. **B.** Deconstruction of a mushroom-derived cell wall extract by the *endo*-β-1,3-glucanase *Cp*Glu16A and the aforementioned chitinases.

| **A.** | **Enzyme(s) in assay** | | |  |  |
| --- | --- | --- | --- | --- | --- |
| **Experiment** | ***Cp*ChiA_N_** | ***Cp*ChiA_C_-CBM5** | ***Cp*ChiB** | **24h** | **48h** |
| **1** | 🗸 | - | - | 0 % | 0 % |
| **2** | - | 🗸 | - | 10.56 ± 1.7 % | 64.03 ± 3.0 % |
| **3** | - | - | 🗸 | 0 % | 0.99 ± 0.17 % |
| **4** | 🗸 | - | 🗸 | 0 % | 0 % |
| **5** | - | 🗸 | 🗸 | 48.84 ± 12.5 % | 91.75 ± 11.4 % |
| **6** | 🗸 | 🗸 | - | 36.96 ± 1.1 % | 93.07 ± 1.7 % |
| **7** | 🗸 | 🗸 | 🗸 | 71.62 ± 4.1 % | 100.00 ± 2.6 % |

| **B.** | **Enzyme(s) in assay** | | | |  |  |
| --- | --- | --- | --- | --- | --- | --- |
| **Experiment** | ***Cp*ChiA_N_** | ***Cp*ChiA_C_-CBM5** | ***Cp*ChiB** | ***Cp*Glu16A-CBM6** | **30min** | **24h** |
| **1** | - | - | - | 🗸 | 4.07 ± 1.9 % | 12.20 ± 4.5 % |
| **2** | 🗸 | 🗸 | 🗸 | - | 8.58 ± 5.8 % | 82.53 ± 10.9 % |
| **3** | 🗸 | 🗸 | 🗸 | 🗸 | 10.84 ± 3.9 % | 100.00 ± 9.1 % |
